# Genetic Architecture of Ischemic Stroke: Insights from Genome-Wide Association Studies and Beyond

**DOI:** 10.3390/jcdd12080281

**Published:** 2025-07-23

**Authors:** Ana Jagodic, Dorotea Zivalj, Antea Krsek, Lara Baticic

**Affiliations:** 1 Department of Family Medicine, Community Health Center Krapina, 49000 Krapina, Croatia; beganovicana05@gmail.com; 2 Faculty of Medicine, University of Rijeka, 51000 Rijeka, Croatia; dorotea.zivalj@gmail.com (D.Z.); antea.krsek@uniri.hr (A.K.); 3 Department of Medical Chemistry, Biochemistry and Clinical Chemistry, Faculty of Medicine, University of Rijeka, 51000 Rijeka, Croatia

**Keywords:** *ANRIL*, genetic risk prediction, genome-wide association studies, *HDAC9*, Ischemic stroke, *PITX2*, polygenic risk score, *SORT1*

## Abstract

Ischemic stroke is a complex, multifactorial disorder with a significant heritable component. Recent developments in genome-wide association studies (GWASs) have identified several common variants associated with clinical outcomes, stroke subtypes, and overall risk. Key loci implicated in biological pathways related to vascular integrity, lipid metabolism, inflammation, and atherogenesis include 9p21 (*ANRIL*), *HDAC9*, *SORT1*, and *PITX2*. Although polygenic risk scores (PRSs) hold promise for early risk prediction and stratification, their clinical utility remains limited by Eurocentric bias and missing heritability. Integrating multiomics approaches, such as functional genomics, transcriptomics, and epigenomics, enhances our understanding of stroke pathophysiology and paves the way for precision medicine. This review summarizes the current genetic landscape of ischemic stroke, emphasizing how evolving methodologies are shaping its prevention, diagnosis, and treatment.

## 1. Introduction

Stroke is a condition of sudden development of focal neurological impairment due to impaired brain blood supply to a portion of the brain. It can be caused by various cerebrovascular pathologies, including ischemic and hemorrhagic types, and is a cause of significant morbidity and mortality worldwide [1]. The global burden of ischemic stroke (IS) has been estimated to rise to 89 per 100,000 people by 2030, with rising rates in women and all age and income groups. Mortality and disability will decline overall but possibly increase in lower-income countries [2]. Ischemic stroke subtypes include cardioembolic (36%), large-artery atherosclerosis (9%), small-artery occlusion (9%), other determined causes (6%), and undetermined causes (39%). Cardioembolic strokes were of higher severity and mortality compared with other subtypes [3]. Elucidation of ischemic stroke’s genetic architecture is important for numerous reasons. Importantly, it untangles complex biological mechanisms for stroke susceptibility, development, and outcome that will differ hugely in different individuals [4].

Understanding genetic predisposing factors, particularly through genome-wide association studies (GWASs), provides the basis for identifying novel molecular mechanisms and candidates for potential intervention [5]. Furthermore, genetic insights support the development of predictive measures like polygenic risk scores that can facilitate early identification of at-risk individuals and guide targeted prevention. In the long term, such information paves the way for precision medicine approaches that can individualize interventions based on a person’s genetic profile and eventually reduce the incidence of stroke and improve clinical outcomes [6]. GWASs have transformed our understanding of the genetic etiology of ischemic stroke by enabling us to discover large numbers of hypothesis-free common single-nucleotide polymorphisms (SNPs) that contribute to disease susceptibility across populations [7].

These studies have revealed several key genomic loci—most notably 9p21, *HDAC9*, and *SORT1*—each of which implicates biological pathways involving vascular integrity, lipid metabolism, atherogenesis, and thrombotic pathways [8,9,10]. Despite these gains, much of the heritable etiology of ischemic stroke remains unexplained, a problem long referred to as “missing heritability” [11]. Sequencing technology developments have highlighted the importance of rare variants, structural variation, and regulatory elements in noncoding regions, whose functions are increasingly recognized as playing a pivotal role in contributing to the understanding of complex traits and bridging genomic variant interpretation gaps [12].

Polygenic risk scores (PRSs) represent a key approach to estimating the genetic susceptibility to IS in a person by summing up the very small effects of numerous frequent variants spread out over the genome [13]. Although their current clinical potential remains modest—particularly in that they need validation in a very diverse range of populations—PRSs have considerable potential to enable early identification of susceptible subjects and target preventive interventions [14].

Aside from GWASs, advanced genomic techniques are increasingly being used to enhance our understanding of the genetic architecture of IS. Functional genomics approaches, such as expression quantitative trait loci (eQTLs) analysis and transcriptomics, are used to examine how individual variants influence gene expression and cellular function [15,16]. Epigenomic studies of DNA methylation and histone modification cast significant illumination on gene–environment interactions and dynamic regulation of stroke-related pathways [17]. Multiomics integration, integrating genomic, transcriptomic, proteomic, and metabolomic data, enables systems-level understanding of how genetic variation is translated into phenotypes at a biological level [18].

**Figure 1:**
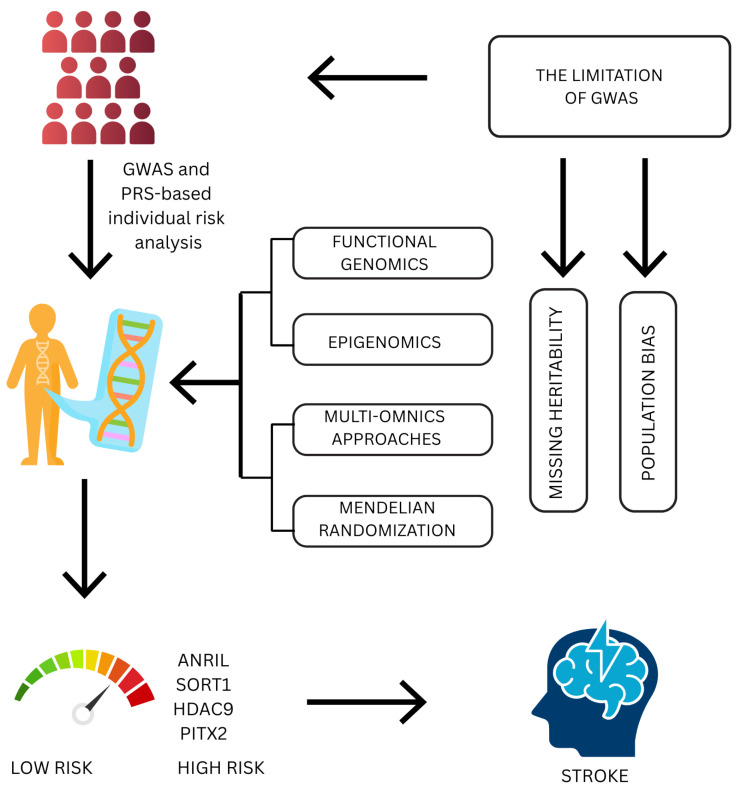
Bridging the gap in stroke genetics: from GWAS to precision medicine. The current framework for comprehending genetic susceptibility to ischemic stroke is depicted in this schematic presentation. Individual risk can be stratified based on common genetic variants, especially within loci like *ANRIL*, *SORT1*, *HDAC9*, and *PITX2*, thanks to genome-wide association studies (GWASs) and polygenic risk score (PRS) analyses. However, population bias and unexplained heritability are two major drawbacks of GWAS approaches. In order to overcome these obstacles, sophisticated techniques like Mendelian randomization, functional genomics, epigenomics, and multiomics integration are being used more and more to clarify underlying molecular mechanisms and make personalized risk prediction and therapeutic targeting possible.

Mendelian randomization further contributes to such strategies by assessing causal associations between modifiable risk factors and stroke outcomes [19]. This review synthesizes current advances in the genetics of IS, with emphasis on results from GWASs and new high-throughput genomic methods (Figure 1). By reviewing landmark findings, current methodological innovations, and their implications for translation, we aim to illustrate how the evolving landscape of stroke genetics is not only deepening biological understanding but also informing the evolution of precision medicine approaches in cerebrovascular disease.

## 2. Genetic Epidemiology of Ischemic Stroke

An estimated 12.2 million new occurrences of IS were reported worldwide in 2019 alone, making it one of the top causes of death and permanent disability worldwide [20]. Although conventional risk factors like smoking, diabetes, and high blood pressure have long been known to exist, there is growing evidence that stroke vulnerability is significantly influenced by genetics. The intricate relationship between environmental exposures and genetic predisposition differs depending on the type of stroke and the patient’s demographics [21].

### 2.1. Heritability Estimates and Family-Based Studies

Animal studies suggest a genetic basis for stroke. Twin studies show that monozygotic twins are more likely to both have strokes than dizygotic twins, and having a family history increases stroke risk (OR 1.65; 95% CI 1.2–2.3). Although stroke often appears in families due to aging, most twin and family studies suggest a stronger genetic influence in patients under 70, with heritability varying by stroke type. Recently, information from GWASs has also been used to evaluate the heritability of stroke [22]. The heritability of IS has been estimated at 37.9%, with notable variation across subtypes: 16.1% for small-vessel disease, 40.3% for large-vessel disease, and 32.6% for cardioembolic stroke. While previously proposed candidate genes did not reach statistical significance after adjustment for multiple testing, three loci previously associated with cardiovascular disease demonstrated significant associations: *ZFHX3* with cardioembolic stroke and *PITX2* and *PHACTR1* with large-vessel stroke [23].

### 2.2. Genetic vs. Environmental Contributions to Ischemic Stroke Risk

Understanding stroke risk requires an understanding of how environmental factors and genetic propensity interact. CADASIL (Cerebral Autosomal Dominant Arteriopathy with Subcortical Infarcts and Leukoencephalopathy) is a hereditary cerebrovascular disorder caused by mutations in the NOTCH3 gene located on chromosome 19 [24]. It is the most prevalent monogenic type of brain small-vessel disease [25]. The impact of the NOTCH3 Arg544Cys (R544C) variation and related vascular risk factors on stroke was investigated in a Taiwanese population study. The study discovered that although the NOTCH3 R544C mutation by itself raised the risk of stroke, carriers of the variant were also much more likely to experience stroke due to modifiable vascular risk factors, such as diabetes mellitus and hypertension [26]. This implies that even in people who have a monogenic tendency, environmental circumstances can affect how a stroke manifests.

### 2.3. Interaction Between Genetic Risk and Lifestyle Factors

A thorough analysis of the combined effects of lifestyle factors and genetic predisposition on the risk of cardiovascular illnesses, including stroke, was reported in *Stroke* (2018). According to the study, lifestyle choices and genetic risk both independently raise the chance of stroke. Nevertheless, there was no discernible relationship between lifestyle factors and genetic risk, indicating that, regardless of genetic predisposition, adopting healthy behaviors can reduce the risk of stroke [27]. Furthermore, the Global Burden of Disease Study 2021 underlined the strong influence of lifestyle variables on stroke risk. According to the study, the main preventable risk factors that contribute to the occurrence of stroke globally are smoking, high blood pressure, and a poor diet that includes a lot of salt and few fruits and vegetables. These results highlight the importance of targeting modifiable behaviors in lowering stroke risk across groups, even while genetic predisposition plays a part in stroke vulnerability [28].

### 2.4. Stroke Subtypes and Their Genetic Distinctions

Large-artery atherosclerosis, cardioembolic stroke, and small-vessel (lacunar) stroke are the three different subtypes of ischemic stroke, which are each distinguished by their hereditary risk profiles and pathogenic mechanisms. Genes linked to atherosclerosis are frequently linked to large-artery atherosclerotic stroke. Genome-wide association studies have connected this subtype to variations in *PHACTR1* and *LDLR*, with *PHACTR1* affecting endothelial dysfunction and vascular remodeling [29].

Family hypercholesterolemia (FH), which is mostly caused by *LDLR* mutations, is significantly linked to early atherosclerotic cardiovascular disease and causes lifelong high low-density lipoprotein cholesterol (LDL-C). Large-artery atherosclerotic stroke was significantly more common in ischemic stroke patients with genetically confirmed FH than in non-FH stroke patients, accounting for almost two-thirds of cases, according to a Taiwanese cohort study. This suggests a major subtype-specific risk [30].

Strong links exist between genetic loci in *PITX2* and *ZFHX3* and cardioembolic stroke, which is mostly caused by atrial fibrillation (AF). Significant associations between the *ZFHX3* variant (rs2106261; OR 1.19, 95% CI 1.11–1.27, and *p* ≈ 10^−7^) and the *PITX2* variant (rs12041331; OR 1.39, 95% CI 1.29–1.49, and *p* ≈ 10^−19^) and cardioembolic stroke—lags that directly overlap with known AF risk loci—were confirmed by a large meta-analysis of GWAS data from over 30,000 cases and 43,000 controls in the METASTROKE consortium [29]. Functional research further demonstrates that decreased *PITX2* expression alters tissue shape and atrial electrophysiology, which increases susceptibility to cardioembolic events and promotes AF [31].

Strong correlations have been found at the *COL4A1*/2, *FOXC1FOXC1*, and *HTRA1* loci—genes essential in preserving the integrity of the cerebral microvasculature—indicating that small-vessel (lacunar) stroke is genetically unique. Mutations in *COL4A1*/2 weaken the cerebral vascular basal lamina, making people more vulnerable to lacunar infarcts and microbleeds [32]. White matter hyperintensities and cerebral small-vessel disease have also been linked to uncommon mutations in *FOXC1FOXC1*, a transcription factor essential for neurovascular development [33]. Additionally, *HTRA1*, linked to familial small-vessel diseases such as CARASIL, is becoming more widely acknowledged for its role in sporadic cerebral small-vessel disease [34].

## 3. Genome-Wide Association Studies in Ischemic Stroke

A GWAS identifies genetic variants linked with ischemic stroke, revealing rare monogenic and common polygenic causes. Polygenic causes account for a significant proportion of cases and enable genetic risk scores for individualized prevention and improved risk stratification [35]. Recent genome-wide association studies have identified many genetic loci associated with IS, casting new light on the pathophysiology and potential prevention and treatment avenues [36].

A single-nucleotide polymorphism is a variation at one spot in the DNA sequence among individuals. SNPs are the most common type of genetic variation and can influence individuals’ development of disease or response to environmental stimuli, drugs, and other therapies. In GWASs, SNPs are used as markers to identify genetic associations with specific traits or diseases [37]. Many SNPs influence IS risk by changing gene expression or protein function [38]. Some lie within regulatory regions, such as promoters or enhancers, and modulate the extent to which a gene is expressed. Others create amino acid substitutions that affect protein structure or function, potentially impairing regular cellular function [39]. Four SNPs near genes *PITX2*, *ZFHX3*, CAV1, and SYNPO2L are significantly linked with atrial fibrillation (AF), a significant stroke risk factor. AF can be caused by genetic variation in these loci by several mechanisms, such as ectopic excitation, inflammation, fibrosis, and cytoskeleton disruption.

Increased circulating cell-free DNA (cfDNA) levels were also linked with prior stroke, suggesting that cfDNA could potentially play a role as a stroke risk biomarker in paroxysmal AF patients [40]. The T allele for the rs11672433 variant in the gene *ANGPTL4* has been associated with a highly diminished risk of atherosclerotic IS. In addition, the interactions of rs11672433 and rs4076317 variants modify stroke risk, suggesting these specific SNPs combined influence susceptibility to disease [41].

GWAS analyses reveal causal links among IS and circulating lipid and blood pressure genetic variants. Lipid metabolism, DNA repair, and neuroprotection genes such as *FURIN*, *ALDH2ALDH2*, and *TOMM40* are implicated. This informs stroke pathogenesis and holds the promise of individualized prevention [42].

Multiple genome-wide association studies have identified genetic loci that are involved in the risk of stroke as well as stroke outcome, focusing on the relationship between genetic susceptibility and recovery patterns. Based on growing evidence, SNPs such as rs12579302 (ATP2B), rs10886430 (*GRK5*), rs55983834 (*SH3PXD2A*), rs2501966 (*CENPQ*), and rs12426667 (*HOXC4*) are associated with genes whose differential expression is critical to stroke outcome [43,44]. Intergenic variants at *HDAC9* (rs2107595) and *BNC2* (rs1487504) highlight the additional genetic contribution to both susceptibility and prognosis.

The ADAM gene family, and particularly *ADAM23* and members of this group, have also been implicated in worse prognosis, again reinforcing the value of these loci as potential biomarkers of stroke outcome and recovery [45]. Early neurological instability after IS is also partially genetic, with shared variants accounting for 8.7% of the variance. Significant genes like *ADAM23* and *GRIA1*, implicated in neuronal excitability, are involved in the excitotoxic role in early post-stroke changes [46].

GWASs play a significant role in uncovering those genetic variants that have an association with IS risk, progression, and outcome. A GWAS enables both rare monogenic and common polygenic contributors to be delineated, making the generation of polygenic risk scores possible, which refine individual risk prediction and stratification. In addition, these findings reveal pathophysiological pathways, such as extracellular matrix disruption and pericyte differentiation, critical for understanding IS [47]. Clinically, this enables the identification of high-risk individuals earlier who can be provided with preventive therapies such as anticoagulation or lipid-lowering therapy, even before symptoms arise [4].

GWAS findings also identify specific biological pathways, including inflammation, lipid metabolism, and vascular remodeling, which can be targeted for the creation of novel therapeutics [48]. For example, *HDAC9* or *ANGPTL4* variants not only identify stroke susceptibility but also inform possibilities for repurposing HDAC inhibitors or enhancing *ANGPTL4*-mediated protection [49,50].

Overall, a GWAS bridges the divide between genetics and personalized medicine, offering a roadmap for precision prevention and treatment in stroke medicine. The primary limitations of GWASs in practice are the need for extremely large sample sizes to uncover the entire genetic architecture of complex traits and a drastic Eurocentric bias that limits generalizability to non-European populations. Further, current genotyping approaches based on microarrays lack the ability to sample all genetic variants, thereby rendering the clinical accuracy less than what can be achieved through whole-genome sequencing [51].

Outside the above limitations, the “missing heritability” issue remains a major concern in GWASs. It refers to the gap between the heritability of complex traits inferred from family studies and the proportion explained by existing genetic variants, suggesting that many causal variants, especially structural and rare variants, are not yet captured by standard approaches [52]. Some of the notable loci implicated in IS by GWASs, including *ANRIL*, *SORT1*, *HDAC9*, and *PITX2*, have been highlighted. These are some of the risk loci that have been uncovered and are featured here due to their strong and replicated associations with stroke risk, subtype specificity, and potential therapeutic relevance. Additional loci such as *FURIN*, PROCR, *GRK5*, *SH3PXD2A*, and LPA also warrant in-depth discussion based on the current literature and biological plausibility [43].

### 3.1. Antisense Noncoding RNA in the INK4 Locus

The 9p21 locus, namely, the Antisense Noncoding RNA in the INK4 locus (*ANRIL*) gene within the INK4/ARF locus, is involved in the regulation of cell proliferation and vascular integrity. Genetic polymorphisms in this locus influence the expression of *ANRIL*, which regulates vascular smooth muscle cell behavior, resulting in the development of atherosclerosis. Dysregulation in *ANRIL* expression and splicing patterns is connected with increased susceptibility to vascular diseases, like stroke and coronary artery disease [53].

The 9p21 locus contributes to stroke vulnerability through the regulation of *ANRIL* gene expression, which is involved in cell proliferation and vascular inflammation. Specific SNPs, such as rs10757274 and rs4977574, have been found to increase ischemic stroke risk, particularly in patients with large-artery atherosclerosis, and have differential effects in different populations [54].

In hypertensive patients with coronary artery disease (CAD), linear *ANRIL* was overexpressed, while circular *ANRIL* decreased, resulting in a decreased circular/linear *ANRIL* ratio. This suggests that linear *ANRIL* promotes atherosclerosis, while circular *ANRIL* may play a protective role in vascular health [55]. Aspirin resistance (AR) in IS is genetically determined, and polymorphisms like rs1330344 in PTGS1 and rs4311994 in ADRA2A influence platelet aggregation. The 9p21 locus, which is linked to vascular inflammation, can also predispose to stroke risk and AR, indicating the necessity for personalized therapy [56]. *ANRIL* plays a role in atherosclerosis via the epigenetic silencing of CDKN2A/B through the recruitment of polycomb repressive complexes, leading to inappropriate growth of vascular smooth muscle cells. Upregulation has independently been implicated as a risk factor for in-stent restenosis, highlighting its involvement in vascular remodeling and neointimal hyperplasia [57].

By sponging miR-399-5p, the lncRNA upregulates FRS2 and initiates the RAS/RAF/ERK pathway to stimulate vascular smooth muscle cell migration and proliferation. It aids in the process of atherosclerosis development and stroke susceptibility enhancement [58]. Aberrant *ANRIL* expression increases the levels of vascular endothelial growth factor (VEGF), activating the NF-κB pathway and promoting angiogenesis and inflammation. This is the cause of atherosclerosis and CAD, with linear *ANRIL* isoforms predicting worse cardiovascular risk and circular isoforms with less oxidative stress [59]. In cerebral venous thrombosis (CVT) patients, plasma levels of *ANRIL* and myocardial infarction-associated transcript (MIAT) were drastically lower than those of healthy controls, both with high diagnostic sensitivity (AUC > 0.98) for lncRNAs as possible blood-based biomarkers of CVT [60].

### 3.2. SORT1

*SORT1* encodes sortilin, a protein involved in lipid metabolism, including the modulation of LDL cholesterol. Since high LDL-C is a cause of atherosclerosis, a common underlying cause of IS, genetic variation in *SORT1* might indirectly influence stroke risk by lipid regulation [61]. Serum sortilin levels were remarkably higher in symptomatic intermediate CAS patients than in asymptomatic individuals, and this reflects a correlation between elevated sortilin and cerebrovascular events, such as stroke or transient ischemic attack (TIA). Sortilin was found to be an independent predictor of symptomatic carotid plaque, and it qualifies as a potential biomarker in assessing the risk of stroke in intermediate carotid artery stenosis (CAS) patients [62].

In new-onset hypertensive patients, plasma sortilin levels were increased, particularly in the subgroup with subclinical carotid atherosclerosis. These positive correlations with IL-6, hsCRP, ET-1, LDL-C, TG, TC, and cIMT and negative correlations with NO and adiponectin suggest that sortilin plays roles in inflammation, endothelial dysfunction, and atherosclerosis [63]. Variants at the 1p13.3 locus are strongly associated with LDL-C, but the effect of *SORT1* on LDL-C is heterogeneous between studies. Exome sequencing in an Amish population identified *SORT1* variants (K302E and Q225H) with effects of increased and lowered LDL-C, respectively, in which humans and mice had different effects. This is suggestive of a complex role for *SORT1* in lipid metabolism [64].

FABPs (fatty acid-binding proteins) play key roles in IS by modulating lipid metabolism and cellular activities in the neurovascular unit. FABP3, FABP4, FABP5, and FABP7 isoforms are activated during the ischemic cascade and contribute to injuring brain tissue. Targeting FABP may open new pharmacological windows for neuroprotective drug therapies for IS [65]. Sortilin interaction with FABP7 increases lipid acquisition and anti-inflammatory gene expression in the brain, a process that is impaired in the apoE4 variant. Sortilin fails to control FABP7 in apoE4 carriers, leading to catabolism and impaired lipid signaling [66]. Serum concentrations of the sortilin-derived propeptide were highly correlated with depression ratings in patients with stroke over time and are suggested to be a possible biomarker for post-stroke depression. This opens up a possible new therapeutic target, which is the drug administration of spadin, an agent that acts against this peptide [67].

### 3.3. Histone Deacetylase 9

Histone deacetylase 9 (*HDAC9*) is an atherosclerosis- and inflammation-promoting enzyme that activates NF-κB pathway signaling. *HDAC9* binds to IKKα/β and deacetylates it, increasing NF-κB and pro-inflammatory gene expression. *HDAC9* risk alleles lead to upregulated immune cell and plaque expression and enhance stroke and other vascular diseases [68]. *HDAC9* promotes neuronal ferroptosis after stroke by increasing HIF-1 activity, which increases the iron importer TfR1, and decreases the Sp1 levels, which inhibits the protective enzyme GPX4. These changes lead to iron overload and lipid peroxidation. Silencing *HDAC9* inhibits these processes, suggesting it is a key player in stroke-induced neuronal damage [69]. The involvement of *HDAC9* in the NOTCH signaling pathway modulates the vascular smooth muscle cell phenotype and promotes atherosclerotic calcification.

Genetic polymorphisms like rs2107595 increase their expression, particularly in carotid and femoral plaques and peripheral blood mononuclear cells, and are implicated in IS susceptibility by enhanced carotid atherosclerosis. Hypomethylation of *HDAC9* in stroke patients also supports its upregulation as a pathogenic process [70]. The inhibition of *HDAC9* may stabilize atherosclerotic plaques by inhibiting inflammasome activation and pyroptosis. Myeloid cell targeting offers a better, more targeted option compared to the more general anti-inflammatory drugs, like IL-1β inhibition [9]. Vascular smooth muscle cells (VSMCs) of carotid plaque have been found to express *HDAC9*, in addition to immune-related genes.

Expression of *HDAC9* modulates macrophage recruitment and may influence plaque instability and progression by modifying the VSMC phenotype and immune cell function [71]. New data finds *HDAC9*’s role in systolic hypertension and Non-ST-Elevation Myocardial Infarction (NSTEMI) risk, with increased correlation to NSTEMI pointing towards non-atherosclerotic mechanisms, like hypertension or arterial calcification. *HDAC9* is confirmed as a regulator of VSMC disease and may represent a novel therapeutic target independent of current therapies. The study also suggests a protective effect on kidney disease, but it is not statistically significant [72].

### 3.4. PITX2 Gene

The *PITX2* gene plays a significant role in susceptibility to ischemic stroke, particularly cardioembolic stroke. A number of *PITX2* variants were related to stroke risk, with nominal significance for ischemic stroke. This makes *PITX2* an excellent candidate for further research and therapeutic purposes [73]. The increased risk of ischemic stroke, including cardioembolic stroke, has been linked with genetic components confirmed through the joint UKBB and TOPMed analysis.

The *PITX2* gene is a candidate to cause susceptibility to stroke, with multiple variants within the gene being nominally associated with ischemic stroke [74]. The *PITX2* gene encodes a transcription factor involved in cardiac development and conduction, particularly atrial function. *PITX2* variants such as rs2200733 and rs6843082 have been reported to elevate the risk of ischemic stroke, especially in people of European ancestry.

The genetic risk appears to vary between populations, perhaps due to ethnic-specific genetic backgrounds and allele frequencies [75]. Variants of the *PITX2* gene have been linked to ischemic stroke risk, particularly in the population of European ancestry. There also exists evidence showing that the rs2634074 variant interacts with sleep duration and thus suggests gene–environment interaction in causing susceptibility to stroke [76]. Genetic variations in the *PITX2* gene, especially in cardioembolic strokes, indicate *PITX2* as a novel target for treatments. Additionally, miRNA-regulated regulatory pathways such as miR-17-92 can offer directions for novel methods of stroke prevention [74].

## 4. Polygenic Risk Scores and Risk Prediction

Genome-wide association studies have identified thousands of genes linked to vascular diseases, including stroke, and PRSs aggregate that genetic information to predict individual disease risk. Polygenic risk scores for IS and intracerebral hemorrhage have a high association with the risk of each event, even after accounting for clinical factors such as hypertension [77]. Polygenic risk scores for IS quantify inherited risk by summing the impact of many common genetic variants. Summing millions of SNPs improves predictive power, as shown by larger hazard ratios and C-indices in large datasets. These scores help with risk stratification, especially within ancestry-matched populations [78].

GWASs provide the basis on which PRSs have been developed, with the identification of genetic variants underpinning IS. PRSs of more genetically heterogeneous GWASs, such as MEGASTROKE, are more likely to generalize across population groups with dissimilar genetic makeups. The implication is crucial in highlighting more extensive representation of GWASs towards guaranteeing fair and consistent applications of PRSs across all sectors [79]. The stroke PRS can predict IS events in an otherwise healthy older population better than most conventional risk factors. Adding the PRS to existing models does enhance prediction modestly, and therefore, further studies are needed to assess its clinical usefulness [80]. Through the combination of 112 risk factor polygenic risk scores (RFPRSs) with disease-specific PRSs, the study developed an improved prediction model, RFDiseasemetaPRS. The model performed superiorly in predicting diseases compared to using disease-specific PRSs alone. The findings suggest the ability of RFDiseasemetaPRS to enhance risk stratification and personalize healthcare strategies [81].

The stroke meta-polygenic risk score (metaGRS) was found to predict ischemic stroke events in a healthy older population, but its addition to standard risk models only improved prediction modestly. Its current clinical utility is limited, and further effort is needed to maximize genetic risk scores and identify the optimal clinical settings for their use [80]. Hypo-HDL-C, a cardiovascular disease and stroke risk marker, is environmental and genetic in nature. A polygenic risk score that included ZPR1_rs3741297, CETP_rs708272, BUD13_rs180327, ALDH1A2_rs588136, and the 11q23.3 haplotype imposed a threefold greater risk of hypo-HDL-C, with diet modifying this risk. The findings provide evidence for the efficacy of SNP-based PRSs in the identification of subjects at higher risk for stroke [82]. Genetic susceptibility to stroke, particularly IL-18 pathway variants, was associated with a 2.5- to 2.9-fold increased risk employing 5-, 6-, and 7-SNP polygenic risk scores.

Lifestyle was not associated individually with stroke risk, but interactions of genes with diet involving PRSs and a high intake of energy, carbohydrates, and branched-chain amino acids (BCAAs) and low n-3 fatty acids had notable influences on stroke chances. These findings point to gene–diet interaction modulating the risk of stroke [83]. A CAD PRS showed strong predictive capacity in younger individuals, enhancing risk stratification and guiding preventive treatment. This illustrates the encouraging clinical utility of PRSs in ischemic stroke, where the identification of genetically at-risk individuals at an early stage can allow targeted preventive measures [84].

Pathway-specific polygenic risk scores assess disease risk based on sets of genes that act in specific biological processes or pathways rather than individual genes. For ischemic stroke, these scores are derived from gene sets defined by databases like Gene Ontology, which group genes based on their functions. In this way, this approach captures a more complete view of genetic risk by accounting for how gene pathways, such as inflammation or clotting, contribute to stroke susceptibility [85]. Pathway-specific polygenic risk scores for IS were independently associated with 3-year all-cause mortality and improved risk prediction over clinical factors. A multivariate model that included 11 PRSs, one of which was linked to endothelial cell apoptosis, had improved prognostic capacity, enabling stratification of patients according to mortality risk [86].

The addition of a cardiovascular disease polygenic risk score (CVD-PRS) to clinical risk assessment appreciably improved the detection of those at high risk for large cardiovascular events, particularly in young patients. This suggests that the integration of genetic data, i.e., CVD-PRS, with standard risk scores can improve prediction and early identification of individuals at higher risk of stroke and other cardiovascular events in daily clinical practice [87]. An integrated polygenic score (PGS) for IS and IS-related disease into clinical risk factors significantly improved risk assessment of IS by enhancing predictability and reclassification of risk. While the inclusion of PGSs provided additional value to the identification of higher-risk individuals, overall clinical utility is limited by modest improvement in predictive performance [88]. Merging PRSs with transcriptomic data through sum transcriptome-polygenic risk scores (STPRSs) significantly improves predictive accuracy for ischemic stroke and its subtypes. It facilitates a better appreciation of the genetic and molecular determinants of stroke and offers the promise of more individualized preventive and treatment strategies. STPRS models predicted IS-related phenotypes better than standard PRSs and defined appropriate genetic markers amenable to clinical practice [89]. PRSs for IS have a number of potential benefits.

Genetic factors also modulate the influence of inflammation, coagulation, and endothelial function on stroke risk. However, it should be emphasized that certain gene variants have been shown to increase or decrease stroke susceptibility depending on the population studied. For example, polymorphisms in the paraoxonase 1 (PON1) gene, which is involved in preventing oxidative damage to lipids, have been associated with both increased and decreased stroke risk in different ethnic groups, highlighting the complexity of genetic–environment interactions [85,86,87,88]. Similarly, variants of the angiotensin-converting enzyme (ACE) gene, known to affect blood pressure regulation and endothelial function, have shown opposing effects on stroke risk in European versus Asian populations [85,86]. These findings emphasize the importance of considering population-specific genetic backgrounds when studying stroke mechanisms and developing targeted prevention strategies.

Genetic risk is fixed throughout life, enabling early recognition of those at increased risk—often before environmental or clinical risk factors develop. This enables active prevention and individualized treatment decisions, for instance, earlier initiation of statin therapy in high-risk individuals [90]. A PRS also has great potential to forecast lifetime risk trajectories and can be applied in conjunction with associated complex conditions like AF, hypertension, and type 2 diabetes, all of which are associated with stroke risk [91]. However, there are still some limitations. The predictive power of a PRS compared to traditional clinical risk factors remains untested, and the models available are largely among people of European descent, reducing accuracy in other groups. Additionally, there is no standard approach to generating a PRS, and no PRS to date has been widely accepted by seminal cardiovascular or stroke guidelines [92]. Whether a universal PRS for atherosclerotic cardiovascular disease is to be used or disease-specific scores, such as those for IS, CAD, or peripheral artery disease (PAD), are to be used is unclear. Finally, the cost-effectiveness and clinical utility of incorporating PRSs into healthcare systems are yet to be established [93].

## 5. Beyond GWASs: Advanced Genomic Approaches

While genome-wide association studies (GWASs) have implicated hundreds of loci in IS, they often do not shed light on the biological mechanism or establish causality. To close this knowledge gap, more recent genomic approaches such as functional genomics, epigenomics, and multiomics integration are being applied with growing regularity to translate association to function [94]. These strategies aim to uncover how genetic variants influence stroke risk through exerting regulatory effects on gene expression, epigenetic modifications, and protein networks.

Furthermore, analytical techniques like Mendelian randomization enable the inference of causal relationships between genetic factors and disease, providing new opportunities for biomarker discovery and therapeutic targeting [95]. While GWASs and PRSs are valuable tools for predicting stroke risk based on genetics, they have significant limitations (missing heritability and population bias). Integrating additional layers of biological information through advanced genomic techniques can improve the understanding and prediction of stroke risk.

### 5.1. Functional Genomics

Large-scale GWASs and sequencing studies, along with advances in functional genomics, have revealed more than 100 common genetic loci linked to the risk of stroke, especially ischemic stroke. Rare variations and structural alterations are still little studied but have the potential to reveal new disease mechanisms, even though the majority of findings involve common variants. In addition to highlighting both shared and ancestry-specific genetic risk factors, cross-ancestry analyses have improved discovery power [36].

When paired with patient-derived induced pluripotent stem cell (iPSC) technology, functional genomics allows for molecular modeling of stroke, providing information on genetic risk and disease mechanisms. Translation to clinical applications is accelerated by iPSC-based models, which combine genome editing, multiomics, and sophisticated 3D systems to enable the exploration of stroke-related pathways and the discovery of possible therapeutic targets [96]. The modeling of particular risk variants in pertinent cell types is made possible by functional validation using patient-derived iPSCs and genome editing tools, such as CRISPR-Cas9 [97].

Noncoding genetic variants typically regulate gene expression and splicing and are a significant source of heritability of complex traits. Splicing QTLs (sQTLs) and expression QTLs (eQTLs) demonstrate the manner in which variation in the genome influences molecular phenotypes and disease susceptibility. Multi-level combinatorial QTL analysis elucidates underlying regulatory mechanisms. Splicing effects and nonprimary signals are critical considerations, illustrating the value of functional genomics multiomics [98]. With the intricate cellular structure of the brain and vasculature, the effects of eQTLs and sQTLs on specific tissues and cell types have come to be understood as essential to comprehending stroke biology. Temporal dynamics are frequently displayed by these regulatory variations, with changes in gene expression occurring during the acute and recovery stages of ischemic injury [99].

Another level of complexity is added by alternative splicing triggered by sQTLs, which may change functional pathways and protein isoforms associated with stroke pathophysiology [100]. Following ischemic injury, transcriptomic analyses such as bulk and single-cell RNA sequencing (scRNA-seq) have shed light on cellular heterogeneity and dynamic changes in gene expression. By identifying discrete cellular subpopulations implicated in tissue repair, BBB disruption, and neuroinflammation, single-cell transcriptomics reveals new candidate genes and pathways that are influenced by genetic variation [101]. Additionally, noncoding RNAs, such as long and microRNAs, help regulate genes and may mediate the effect of genetic variations on the risk of stroke [102]. Technological research and the identification of possible therapeutic targets are made easier by functional validation via gene editing in iPSC-derived models and organoids [103]. These developments open the door for ischemic stroke precision medicine approaches that use functional genomics for personalized diagnosis and care.

### 5.2. Epigenomics

Without changing the DNA sequence, epigenetic modifications are reversible and heritable changes that control gene expression [104]. Epigenetic processes like DNA methylation, histone modifications, and noncoding RNAs have become crucial regulators of pathophysiological processes in ischemic stroke [105].

DNA methylation can either activate or silence genes related to oxidative stress, inflammation, vascular function, and neuronal survival. It usually takes place at CpG sites. Numerous investigations have shown that stroke patients exhibit abnormal methylation patterns, linking genes linked to immunological response, neuroprotection, and endothelial dysfunction [106]. Acetylation, methylation, and phosphorylation are examples of histone modifications that change the accessibility of genes and the structure of chromatin, which affects transcriptional programs that are essential for ischemic injury and recovery. For instance, by modifying the pathways of inflammation and apoptosis, HDAC inhibitors have demonstrated neuroprotective effects in experimental stroke models [107].

Furthermore, the effect of environmental risk factors like diet, smoking, and high blood pressure on the risk of stroke may be mediated by epigenetic modifications. Additionally, these mechanisms show promise as targets for new therapeutic approaches that aim to alter gene expression profiles in order to improve neurovascular recovery following ischemia. The intricate epigenomic landscape of stroke is still being uncovered by current research, which could have implications for precision medicine and the creation of biomarkers [108].

### 5.3. Multiomics Approaches

Integrative multiomics techniques are required due to the complex nature of ischemic stroke pathogenesis, which involves numerous molecular layers and pathways. To create a thorough molecular profile of disease states, multiomics integrates information from genomics, transcriptomics, proteomics, metabolomics, and epigenomics [109]. The identification of important regulatory networks and the interplay between genes, proteins, and metabolites that underpin stroke risk, progression, and recovery is made possible by this systems biology approach [110].

Information on inherited genetic variants and mutations linked to stroke susceptibility can be found in genomic data. Transcriptomic analyses highlight active molecular responses by revealing changes in gene expression in blood cells or brain tissue during and after stroke. Changes in protein abundance and post-translational modifications associated with neuroinflammation, thrombosis, and repair mechanisms are identified by proteomic profiling [111].

By describing the tiny molecules involved in oxidative stress and energy metabolism, metabolomics adds another layer. Combining these datasets makes it easier to find new biomarkers for prognosis and early diagnosis. It also maps dysregulated pathways to find possible therapeutic targets [112].

For example, endothelial dysfunction pathways and signaling cascades linked to inflammation have been identified by combined omics analyses as being crucial in IS [113]. Using integrated bioinformatics analyses, important genes related to iron metabolism and inflammation in IS were found. Important biological functions are highlighted by pathway and protein interaction results, and the immune environment of the disease is clarified by immune profiling. These results lay the groundwork for upcoming clinical and experimental confirmation of therapeutic targets and mechanisms [114]. In order to analyze and interpret multiomics data and advance stroke research toward personalized medicine, advances in computational biology and bioinformatics are essential [115].

### 5.4. Mendelian Randomization

Mendelian randomization (MR) is an analytical technique that infers causal relationships with disease outcomes by using genetic variants as proxies (instrumental variables) for modifiable risk factors [116]. By taking advantage of the random assignment of alleles at conception, which resembles a natural randomized controlled trial, MR overcomes the drawbacks of conventional observational studies in ischemic stroke research, including confounding and reverse causation [117]. Mendelian randomization calculates the direct causal effects of exposures on stroke risk by identifying genetic variants that are highly correlated with exposures, such as blood lipid levels, blood pressure, body mass index, or inflammatory markers.

This method has confirmed targets for intervention by elucidating the causal roles of hypertension and LDL-C as significant contributors to stroke [118]. Moreover, MR studies have been employed to assess possible new risk factors and biomarkers, offering proof to rank targets for prevention and medication development [119]. For example, MR analyses have indicated that certain coagulation factors and elevated C-reactive protein have a causal effect on the risk of stroke [120].

Although MR has limitations, such as the requirement for reliable instrumental variables and possible pleiotropy, its robustness is increased by continuous methodological developments [121]. To sum up, MR is an effective technique for proving causation in stroke genetics and going beyond association, which helps direct translational studies and precision medicine projects [118].

## 6. Biological Mechanisms and Pathways

### 6.1. Inflammation, Coagulation, and Endothelial Dysfunction as Key Pathways in Ischemic Stroke

Beyond their role in the progression and outcomes of IS, inflammation, coagulation, and endothelial dysfunction are also critical contributors to the risk and onset of stroke [1,2,3,4,5,6,118,119,120]. Chronic low-grade inflammation and endothelial activation promote the development of atherosclerotic plaques by enhancing leukocyte adhesion, oxidative stress, and lipid accumulation in arterial walls [118,119]. Over time, this pro-inflammatory and prothrombotic environment destabilizes plaques, increasing the risk of plaque rupture and subsequent thrombus formation, which is a major cause of atherothrombotic stroke. In cardioembolic stroke, systemic inflammation and hypercoagulability often accompany conditions like atrial fibrillation, leading to the formation of cardiac thrombi that may embolize to cerebral vessels [120,121]. Similarly, in cerebral small-vessel disease (cSVD), sustained endothelial dysfunction contributes to blood–brain barrier disruption, impaired autoregulation, and vascular stiffening, all of which elevate the risk of lacunar infarctions. Together, these pathways not only influence stroke severity after onset but also represent fundamental mechanisms driving stroke susceptibility across different subtypes [2,120].

An important component of the pathobiology of stroke, a fatal condition that ranks second in the world in terms of causes of mortality after heart failure, is inflammation. All phases of the ischemic cascade, from the early harmful events brought on by artery obstruction to the late regeneration mechanisms that underlie post-ischemic tissue repair, involve inflammatory signals [122]. Damaged neurons, astrocytes, and endothelial cells produce danger-associated molecular patterns (DAMPs), such as HMGB1 and ATP, following the beginning of cerebral ischemia. These DAMPs trigger the production of pro-inflammatory cytokines (including IL-1β and TNF-α) and chemokines that worsen neuronal damage and interfere with the blood–brain barrier by activating pattern-recognizing receptors, including Toll-like receptors, on microglia and invading immune cells [123]. Thromboinflammation, the interplay between clotting and inflammation, drives ischemic stroke progression by impairing microcirculation and causing I/R injury.

**Figure 2:**
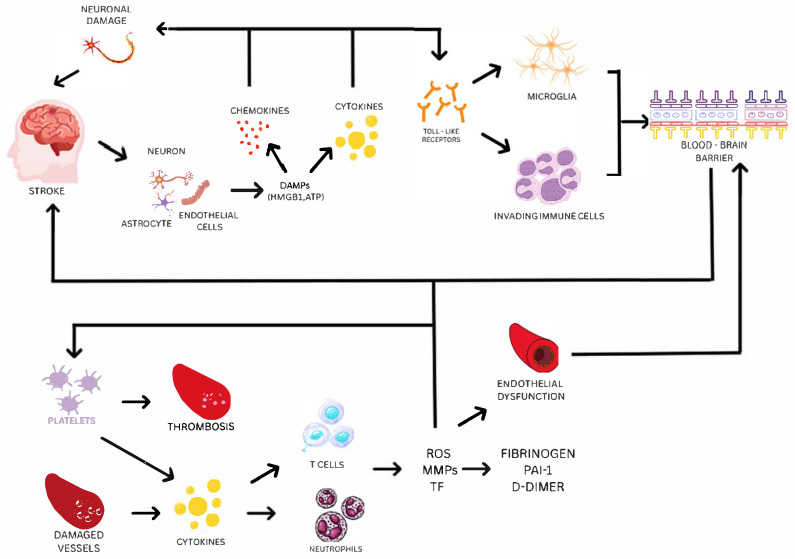
Endothelial dysfunction, coagulation, and inflammation in the pathophysiology of ischemic stroke. Damaged neurons, astrocytes, and endothelial cells release DAMPs like ATP and HMGB1 after an ischemic stroke. These DAMPs activate Toll-like receptors on microglia and immune cells that have infiltrated the body. This exacerbates neuronal damage and compromises the BBB by causing the production of pro-inflammatory cytokines (e.g., IL-1β and TNF-α) and chemokines. Platelet activation, microthrombus formation, and the recruitment of neutrophils and T cells all contribute to the development of thromboinflammation. These processes release TF, MMPs, and ROS, which worsen inflammation and further damage the endothelium. A major factor in the development of stroke is endothelial dysfunction, which causes oxidative stress, decreased vasodilation, leukocyte adhesion, and elevated vascular permeability. These mechanisms create a vicious cycle of thrombosis and inflammation, which exacerbates neuronal damage, BBB disruption, and microvascular occlusion. Elevated levels of fibrinogen, plasminogen activator inhibitor-1 (PAI-1), and D-dimer are biomarkers of this pathological cascade.

During a stroke, platelets rapidly activate and form clots, while damaged vessels and platelets release inflammatory signals that attract immune cells (neutrophils and T cells). Although these cells aid repair, their overactivation releases harmful reactive oxygen species (ROS), matrix metalloproteinases (MMPs), and tissue factor (TF), which worsen inflammation and further activate platelets, creating a vicious cycle of clotting and inflammation [124]. Platelets actively regulate adaptive as well as innate immunity, along with their function in remodeling the vascular system and maintaining vascular integrity. By altering the migration of leukocytes and cytokine signaling, they greatly enhance thrombotic and inflammatory reactions and contribute to the inflammatory environment of stroke [125]. The coagulation cascade is triggered concurrently. One of the main mechanisms in stroke pathogenesis is thromboinflammation, which refers to the interconnectedness of inflammation and thrombosis. Along with inflammatory cells like neutrophils and T cells, activated platelets create microthrombi and release MMPs, TF, and ROS, all of which exacerbate endothelial damage and obstruct the microvasculature [124]. The importance of systemic prothrombotic states has been supported by the discovery of coagulation dysfunction markers in ischemic stroke patients, particularly in small-vessel disease. These markers include increased fibrinogen, plasminogen activator inhibitor-1 (PAI-1), and D-dimer [126], as presented on Figure 2.

One of the main causes of ischemic stroke’s onset and progression is endothelial dysfunction. Vasoconstriction, oxidative stress, leukocyte adhesion, and a prothrombotic setting are all encouraged by dysfunctional endothelium. Additionally, ischemic injury breaks down the BBB tight connections, allowing immune cells and dangerous plasma components to move into the brain parenchyma [127]. The ischemic environment during the subacute period after a stroke causes angiogenesis, which is primarily mediated by endothelial cells under the influence of nitric oxide (NO), VEGF, and hypoxia-inducible factors (HIFs). But if these repair mechanisms are dysregulated, they can also result in aberrant neovascularization and more BBB disruption [128]. Furthermore, a decrease in the level of nitric oxide (NO) bioavailability, which results in poor vasodilation and elevated vascular tone, is a hallmark of endothelial dysfunction. This imbalance increases the incidence of thrombotic events and aids in the advancement of atherosclerosis [127].

Recent studies have highlighted the critical role of oxidative stress in exacerbating endothelial dysfunction and BBB disruption during ischemic stroke. Oxidative stress leads to the production of ROS, which damages endothelial cells and compromises the integrity of the BBB. This damage facilitates the infiltration of immune cells into the brain parenchyma, amplifying neuroinflammation and neuronal injury [129].

In addition to ROS, inflammatory cytokines such as interleukin-1β (IL-1β) and tumor necrosis factor-alpha (TNF-α) contribute to endothelial dysfunction by promoting leukocyte adhesion and increasing vascular permeability. These cytokines activate signaling pathways that lead to the expression of adhesion molecules on endothelial cells, facilitating the recruitment of immune cells to the site of injury [130]. Targeting these inflammatory pathways presents a promising therapeutic strategy to mitigate endothelial dysfunction and BBB disruption in ischemic stroke. Recent research has focused on developing inhibitors that block the activity of key inflammatory mediators, aiming to preserve endothelial integrity and reduce neuroinflammation [131].

### 6.2. How GWAS Findings Point to New Insights in Stroke Biology

Our understanding of ischemic stroke has been greatly expanded by recent developments in the GWAS, which have identified new genetic loci associated with stroke vulnerability and its subtypes. These results draw attention to important biological processes that together contribute to the pathophysiology of stroke, including lipid metabolism, vascular integrity, inflammation, and coagulation. The role of lipid metabolism pathways is one important finding from the GWAS. The significance of cholesterol modulation in cerebrovascular disease is highlighted by the numerous associations between ischemic stroke risk and variations in genes, like *PCSK9*, *LDLR*, and APOE. For instance, *PCSK9* mutations affect LDL-C levels, and loss-of-function mutations are associated with a lower risk of stroke, indicating that *PCSK9* inhibitors may be used therapeutically to prevent strokes [29]. Additionally, GWAS meta-analyses have shown lipid-related genes that influence atherosclerosis and ischemic stroke subtypes, highlighting common pathways [132]. One of the most significant areas highlighted by GWAS findings is lipid metabolism. Numerous associations between ischemic stroke risk and variations in genes like *PCSK9*, *LDLR*, and *APOE* underscore the importance of cholesterol modulation in cerebrovascular disease. For instance, loss-of-function variants in *PCSK9*, such as rs11591147, have been shown to lower LDL-C levels and are associated with a reduced risk of ischemic stroke. This strongly supports the potential of *PCSK9* inhibitors as a preventive therapy for stroke, particularly in individuals with hyperlipidemia [133].

Mendelian randomization studies based on GWAS data have strengthened the causal link between LDL-C/apoB and ischemic stroke subtypes. In a 2020 MR analysis, genetically elevated LDL-C and apoB were specifically tied to increased risk of large-artery stroke (LAS), while *PCSK9*-mediated LDL-C reduction showed protective effects across all ischemic stroke types [134]. Adding to this, the INTERSTROKE study’s worldwide analysis revealed that the apolipoprotein B/A1 ratio was a more reliable and powerful indicator of IS than conventional lipid markers, like LDL-C or non-HDL-C. The study found that apoB/A1 substantially outperformed the LDL-C/HDL-C ratio, with each standard deviation rise linked to a 38% increased risk of stroke [135]. These results emphasize the significance of apoB-related pathway targeting in stroke prevention and the significance of apolipoprotein balance in cerebrovascular risk stratification. Furthermore, fine-mapping efforts via deep-coverage sequencing confirmed that the *PCSK9* variant rs11591147 lowers LDL-C and confers stroke protection, reinforcing its status as a prime therapeutic target [133]. Vascular-specific genes have been discovered by GWASs, along with lipids. *PPAP2B* (LPP3) was discovered as a result of ischemic stroke and CAD. Under hemodynamic stress, in a GWAS, a mechanosensitive gene whose expression is regulated by endothelial flow via miR-92a is essential for preserving the integrity of the endothelium barrier [136]. This locus illustrates how flow-responsive genes help maintain vascular homeostasis and may influence stroke risk. Post-2018 GWASs and multiomics analyses have implicated extracellular matrix (ECM) pathways in stroke susceptibility. A comprehensive 2023 review by Chmelova et al. identified upregulation of MMPs and ADAMTS (a disintegrin and metalloproteinase with thrombospondin motifs) in ischemic injury, particularly in aged brains. These findings suggest that enhanced ECM degradation contributes to BBB breakdown and worsened stroke outcomes [137]. According to the study, worsening stroke outcomes, increased vascular permeability, and BBB disintegration are all caused by increased ECM degradation. In stroke, dysregulated ECM remodeling amplifies inflammatory cell infiltration and reduces the function of the neurovascular units.

Additionally, a 2021 trans-ethnic GWAS meta-analysis of lacunar stroke verified that genetic loci linked to lacunar stroke are abundant in pericyte differentiation (FOXF2 and GPR126), TGF-β signaling (*HTRA1*), and ECM disruption pathways (*COL4A2*, LOX, *SH3PXD2A*, GPR126, and *HTRA1*) [138]. This data demonstrates that the pathophysiology of lacunar stroke is fundamentally influenced by ECM deterioration and associated pericyte and TGF-β-driven microvascular dysfunction rather than being secondary consequences. To maintain microvascular integrity and lessen ischemia injury, these findings collectively emphasize ECM remodeling as a key mechanism in stroke biology, pointing to possible therapeutic targets in MMP inhibition, pericyte stabilization, and TGF-β signaling modulation.

Basal lamina, tight junctions, pericytes, and astrocytic components of the neurovascular unit (NVU) are now recognized by functional genomic frameworks as genetic mediators of stroke. For instance, functional validation using iPSCs demonstrates that several variations generated from GWASs compromise the integrity of the BBB and NVU elements [96]. More than 50 GWAS loci converge on pathways involving ECM, pericyte differentiation, TGF-β signaling, basal lamina integrity, and tight-junction homeostasis, indicating their dysregulation in microvascular permeability and ischemia, according to a thorough review of the genetics of cerebral small-vessel disease [139]. Endothelial cells with stroke risk variants exhibit reduced tight-junction protein expression (e.g., CLDN5 and OCLN) and altered responses to inflammatory signals, which degrade barrier function, according to recent iPSC-derived models [96]. NVU dysfunction in high-burden small-artery illness is characterized by pericyte dedifferentiation, astrocytic activation, and endothelial leakiness, according to single-cell transcriptome analysis of human post-stroke tissue [140]. *ZFHX3* (16q22) and *PITX2* (4q25) variants are two of the most potent GWAS indicators for AF and cardioembolic stroke [29,141]. According to functional follow-up research, *PITX2* affects endothelial cells’ Wnt/β-catenin signaling, a pathway necessary for the integrity of the blood–brain barrier, indicating that cardiac arrhythmia and NVU dysfunction share a molecular basis [142]. Promising methods to preserve microvascular health and lessen ischemia injury include focusing on these pathways, which include restoring tight-junction integrity, maintaining pericyte-basal lamina connections, and modifying Wnt/β-catenin and TGF-β signaling.

### 6.3. Key Genes and Their Involvement in Stroke Risk Pathways

Large-scale genetic investigations conducted in recent years have revealed how variations in important genes map onto several biochemical pathways that modulate the risk of stroke.

#### 6.3.1. PCSK9

Proprotein convertase subtilisin/kexin type 9 (*PCSK9*) has become a key modulator of plasma LDL-C levels and stroke risk in the lipid metabolism pathway. Alleles that cause loss of function, most prominently the missense variant rs11591147, interfere with *PCSK9*’s capacity to bind and guide LDL receptors (*LDLRs*) toward lysosomal degradation. Consequently, the hepatocyte surface’s *LDLR* density rises, improving the removal of circulating LDL-C and lowering serum levels by as much as 30% in heterozygous carriers. Mendelian randomization analyses show that people with these *PCSK9* loss-of-function alleles have a roughly 25–30% lower risk of ischemic stroke than people without them. This is a potent human-genetic “proof of mechanism” that has influenced the creation and use of *PCSK9*-inhibiting monoclonal antibodies and small interfering RNA therapies in clinical settings [133].

#### 6.3.2. APOE

In comparison to ε3 homozygotes, the ε4 allele increases the risk of IS (particularly small-vessel stroke) by approximately 37% due to decreased lipoprotein clearance at the neurovascular interface; homozygous ε4 carriers have a risk that is nearly doubled, suggesting that *APOE* plays a role in cerebral lipid homeostasis and artery integrity [143].

*PPAP2B*, which encodes lipid phosphate phosphatase 3 (LPP3), was first implicated in stroke risk by the MEGASTROKE consortium’s multi-ancestry GWAS of over 520,000 individuals, mapping a susceptibility locus to chromosome 1p32.2 [29]. *PPAP2B* encodes LPP3, which degrades extracellular lysophosphatidic acid (LPA) to prevent its pro-inflammatory and permeability-increasing effects on the endothelium. Under laminar shear, KLF2 maintains LPP3 expression, preserving barrier integrity. In contrast, disturbed flow upregulates miR-92a, which degrades *PPAP2B* mRNA, reducing LPP3 levels and allowing LPA to accumulate. Elevated LPA then induces endothelial contraction and intercellular gaps, compromising the blood–brain barrier and increasing ischemic stroke risk. These flow-dependent effects were demonstrated in human endothelial cells and animal models, linking GWAS-identified *PPAP2B* loci to vascular homeostasis [136].

#### 6.3.3. ARHGEF10

Candidate-gene analysis in a northern Han Chinese community initially linked the gene *ARHGEF10*, which codes for a guanine nucleotide exchange factor unique to RhoA, to the risk of IS. Using dominant, recessive, and additive genetic models, researchers discovered that carriers of the rs2280887 GG genotype had approximately 1.8 times increased odds of ischemic stroke [144]. Vascular permeability is increased by *ARHGEF10*’s biological activation of RhoA in endothelial cells, which results in the production of contractile stress fibers, remodeling of the actin cytoskeleton, and the opening of intercellular gaps. Although this connection has not yet been confirmed in large multi-ethnic GWASs, it points to a mechanism by which variations in *ARHGEF10* may make people more susceptible to stroke by impairing the integrity of the BBB through endothelial hyperpermeability [145].

#### 6.3.4. COL4A1/2

The α1 and α2 chains of type IV collagen, which are essential parts of the cerebral microvascular basement membrane, are encoded by the *COL4A1* and *COL4A2* genes. Inherited cerebral small vascular disorders (cSVDs), including HANAC and PORENCEPHALY, are caused by rare mutations in these genes and are typified by lacunar infarcts, microbleeds, and arterial fragility. Recent extensive genetic studies have demonstrated that common variations at the *COL4A1*–*COL4A2* locus also have a major role in white matter hyperintensities (WMHs), which are radiological indicators of cSVD, and sporadic lacunar stroke [32]. Twelve genome-wide significant loci, including *COL4A2*, were found in a meta-analysis that combined multi-ancestry GWASs and MRI-confirmed lacunar stroke cases (a total of 7338 cases and 254,798 controls). Pathway analysis revealed that ECM dysfunction is a crucial biological mechanism implicated in the pathogenesis of lacunar stroke. This demonstrates that *COL4A1*/2 variations promote small-vessel ischemia injury, weaken the basal lamina, and disrupt the BBB [138].

#### 6.3.5. IL6-R

Genetically downregulated IL-6 signaling is linked to a 12% decreased incidence of ischemic stroke and comparable decreases in coronary artery disease, according to a Mendelian randomization analysis employing genetic proxies for IL-6R inhibition (such as the Asp358Ala variant, rs2228145). In cerebrovascular disease, this offers strong human genetic evidence supporting IL-6R as an effective anti-inflammatory target [146].

#### 6.3.6. F11

A crucial part of the intrinsic coagulation cascade, factor XI is encoded by the *F11* gene. Variations in its genetic makeup have now been causally connected to ischemic stroke, particularly the cardioembolic stroke subtype. A 1-standard deviation (SD] increase in genetically proxied factor XI levels is associated with a 31% increased risk of cardioembolic stroke, according to Mendelian randomization analyses using genome-wide protein quantitative trait loci (pQTLs] from large-scale cohorts, including GIGASTROKE data, which included approximately 62,000 IS cases [147]. Early-phase trial data (e.g., milvexian) showing decreased IS risk without increased hemorrhage rates support the possible clinical use of factor XI inhibitors as antithrombotic agents with a potentially improved bleeding profile, which is further supported by this strong genetic evidence [148].

## 7. Clinical Implications and Applications

Clinical approaches for risk assessment, prevention, and treatment are being impacted more and more by the expanding knowledge of the genetic architecture of ischemic stroke, which is mostly being driven by GWASs. Several applications are emerging that bring stroke care closer to precision medicine, even though implementation in routine clinical practice is still limited [149]. By identifying people with heightened inherited susceptibility, genetic profiling has the potential to improve conventional risk prediction. People can be categorized according to their genetic susceptibility to stroke using a PRS, which combines the effects of several common variants [14]. Rare monogenic causes of stroke, such as mutations in NOTCH3 (linked to CADASIL), *COL4A1*, or *HTRA1*, may also be found with genetic testing [5]. These mutations are especially important in patients who have early-onset or familial stroke. Furthermore, by directing more aggressive intervention in genetically high-risk individuals, even in the absence of traditional risk factors, a wider use of genetic data may aid in the improvement of secondary prevention strategies [150,151]. Another promising use in stroke care is pharmacogenomics, which involves customizing medication therapy based on genetic variation. While polymorphisms in CYP2C19 impact the efficacy of clopidogrel, an antiplatelet medication frequently used in secondary prevention, variations in genes like CYP2C9, VKORC1, and CYP4F2 can have a substantial impact on warfarin metabolism and dosage [152]. By identifying patients at risk of treatment failure or adverse events, genotyping for these variants may enable more individualized and successful therapy. Despite being more frequently employed in cardiology, such testing is becoming more widely acknowledged for its possible significance in cerebrovascular disease [153]. Even with these encouraging advancements, a number of obstacles still exist. The predictive value of current PRSs is only moderate, and the generalizability of many findings is limited because they are primarily derived from populations with European ancestry [154]. Genetic testing also has social, legal, and ethical ramifications, especially when it comes to patient consent, insurance discrimination, and incidental findings. To fully achieve the promise of personalized stroke care, more research in a variety of populations and validation of genetic tools in prospective clinical studies are necessary [155]. Table 1 summarizes key genetic markers implicated in ischemic stroke, emphasizing their functional roles and clinical and pathophysiological mechanisms.

## 8. Challenges and Future Directions

Recent advances in stroke genetics have enhanced our understanding of the molecular mechanisms of ischemic stroke, but several important challenges remain to be overcome. These include the need for larger population-based inclusion in studies of issues of clinical utility, ethical issues, and linkage to new technologies [149]. A significant problem in contemporary genetic research is the overabundance of individuals with European ancestry. This disproportionate focus shifts the center of gravity away from generalizability and could obscure population-specific genetic variants that are essential for assessing risk and discovering therapies [156]. Initiatives to shift the focus toward underrepresented populations are necessary to create more precise genetic models.

Projects like the SIREN Project in Africa, as well as the expanding biobank collaborations in Asia and Northern Europe, are starting to address this problem [157]. Increased investment in globally representative cohorts will reveal novel loci, refine polygenic risk prediction tools, and ensure equitable application of future therapies [158]. Translation of the genetic discoveries into the clinic remains a major challenge. Even with genome-wide studies and sequencing research that have revealed numerous stroke-associated loci, the translation of all this information into daily patient practice remains small [159]. Polygenic risk scores have been promising in quantifying individual susceptibility but require further refinement and cross-validation in various groups [160].

Moreover, the ability to connect genetic information with treatment plans that are tailored to individual patients, such as custom antiplatelet or anticoagulant therapies, will only be possible after the development of reliable, clinically validated frameworks and affordable healthcare system pathways [161]. Aside from technological advancements, social and ethical considerations are underdeveloped. Ensuring privacy, informed consent, and appropriate data handling becomes more difficult as genetic testing gets adopted on a larger scale [162]. Genetic counseling helps patients manage their expectations in a way that minimizes anxiety and stigma. At the same time, the increasing curiosity about gene-editing technologies like CRISPR-Cas9 poses serious concerns about safety, use, equitable access, and overall social acceptability in the context of neurodegenerative diseases [163]. Several variants associated with stroke are located within noncoding sequences of the genome, which adds complexity to the interpretation of gene-targeting approaches, emphasizing the need for robust ethical consideration [4].

Developing new technologies is having a greater impact on research related to stroke genetics. The use of artificial intelligence and machine learning is especially useful for dealing with the intricate, multi-gene factors associated with ischemic stroke [164]. Unlike individual variant-focused models, traditional models toil over singular patterns. Machine learning provides a huge advantage over traditional approaches by analyzing large datasets to identify complex patterns. Multilayered patterns improve predictive power while revealing deeper insights into gene–environment interactions [165].

Moreover, machine learning aids in the integration of multiomics (genomics, transcriptomics, proteomics, and metabolomics) to unveil the biological frameworks that underlie stroke and its recovery. All of these approaches can shape future cancer-based research and clinical integration [166]. Together, they are the future of ischemic stroke genetics. Meeting them will require concerted international effort, sensitive thinking about ethics, and consistent innovation in all scientific and clinical disciplines.

## 9. Conclusions

Both monogenic and polygenic factors that affect disease susceptibility, pathogenesis, and clinical outcomes are part of the complex genetic architecture of ischemic stroke. Our knowledge has been greatly increased by developments in GWASs, which have identified important loci linked to vascular remodeling, lipid metabolism, inflammation, and atrial electrophysiology, including *ANRIL*, *SORT1*, *HDAC9*, and *PITX2*. Despite these advancements, a significant amount of heritability cannot be explained, underscoring the significance of structural genomic elements, gene–environment interactions, rare variants, and epigenetic mechanisms. Although population bias and a lack of validation in diverse cohorts continue to limit the clinical utility of PRSs, they offer promising tools for personalized risk stratification and preventive strategies. Mendelian randomization and integrative methods that integrate transcriptomics, epigenomics, and proteomics are opening the door to precision medicine in stroke. In summary, continued efforts to describe the genetic landscape of ischemic stroke are essential for creating personalized, genetically based predictive, preventive, and therapeutic approaches in addition to expanding our biological understanding.

## Figures and Tables

**Table 1:** Key genetic markers implicated in ischemic stroke: functional roles and clinical and pathophysiological mechanisms.

Gene	Function/Role	Stroke Association	Mechanism/Molecular Pathway	Reference
*PHACTR1*	Regulates actin cytoskeleton and endothelial function	Associated with large-artery atherosclerotic stroke	Involved in vascular remodeling and endothelial dysfunction	[29]
*LDLR*	Low-density lipoprotein receptor involved in cholesterol metabolism	Increases risk of large-artery stroke (especially in familial hypercholesterolemia)	Modulates circulating levels of cholesterol; influences atherosclerosis	[30]
*ZFHX3*	Transcription factor involved in cardiac conduction	Strongly associated with cardioembolic stroke	Implicated in atrial fibrillation and atrial remodeling	[29]
*PITX2*	Homeobox transcription factor involved in cardiac development	Strong association with cardioembolic stroke	Alters atrial electrophysiology and structure; influences risk of atrial fibrillation	[31]
*COL4A1/COL4A2*	Encode type IV collagen; key in vascular basement membrane	Associated with lacunar stroke and cerebral microbleeds	Disrupts blood–brain barrier and vessel integrity	[32]
*FOXC1*	Transcription factor involved in neurovascular development	Linked to white matter lesions and small-vessel disease	Influences brain vasculature and white matter health	[33]
*HTRA1*	Serine protease; regulator in familial cerebral small-vessel disease	Implicated in both familial and sporadic lacunar stroke	Modulates extracellular matrix and TGF-beta signaling	[34]
*ANGPTL4*	Angiopoietin-like protein involved in lipid metabolism	Associated with reduced risk of atherosclerotic stroke	Regulates lipid levels and vascular inflammation	[41]
*FURIN*	Protease involved in protein processing and neuroprotection	Implicated in ischemic stroke susceptibility	Involved in lipid metabolism, neuronal repair pathways	[42]
*ALDH2*	Enzyme involved in oxidative stress response and DNA repair	Associated with ischemic stroke	Detoxifies reactive aldehydes and reduces neuronal injury	[42]
*TOMM40*	Mitochondrial membrane protein	Associated with ischemic stroke	Involved in neuroprotection and mitochondrial integrity	[42]
*ATP2B1*	Calcium transport gene	Influences stroke outcome	Regulates vascular tone and blood pressure	[43]
*GRK5*	G protein-coupled receptor kinase	Linked to ischemic stroke prognosis	Influences cardiovascular remodeling and inflammation	[43]
*SH3PXD2A*	Cell migration and matrix remodeling gene	Implicated in stroke recovery	Modulates extracellular matrix degradation and vascular repair	[43]
*CENPQ*	Centromere protein Q	Associated with stroke recovery outcomes	Regulates cell cycle and genomic stability	[43]
*HOXC4*	Transcription factor	Linked to stroke prognosis	Regulates developmental genes involved in repair processes	[44]
*BNC2*	Transcription factor	Associated with stroke susceptibility and outcome	Modulates gene expression related to inflammation	[45]
*ADAM23*	Involved in neuronal adhesion and excitability	Linked to poor stroke outcome	Influences synaptic function and excitotoxic damage	[46]
*GRIA1*	Glutamate receptor subunit	Associated with early neurological instability	Mediates excitotoxicity post-stroke	[46]
*ANRIL*	Long noncoding RNA regulating cell proliferation and vascular health	Increases ischemic stroke risk (especially large-artery subtype)	Influences vascular smooth muscle cell growth, inflammation, and atherosclerosis	[53,54,55,56,57,58,59,60]
*SORT1*	Encodes sortilin; involved in lipoprotein metabolism	Increases ischemic stroke risk	Modulates cholesterol levels, inflammation, and endothelial function	[61,62,63,64,65,66,67]
*HDAC9*	Histone deacetylase influencing inflammation and vascular remodeling	Strong association with ischemic stroke risk and progression	Activates NF-kappaB; enhances atherosclerosis, ferroptosis, and plaque instability	[68,69,70,71,72]
*PPAP2B*	Encodes lipid phosphate phosphatase 3; endothelial barrier regulator	Associated with ischemic stroke	Maintains blood–brain barrier integrity by degrading lysophosphatidic acid	[136]
*ARHGEF10*	Rho guanine nucleotide exchange factor	Linked to ischemic stroke risk in Han Chinese population	Alters endothelial permeability via actin cytoskeleton remodeling	[144,145]
*APOE*	Lipid transporter in the brain and vasculature	Increases risk of small-vessel stroke	Impairs lipid clearance and damages blood–brain barrier	[143]
*PCSK9*	Modulates LDL receptor degradation	Loss of function reduces ischemic stroke risk	Lowers LDL cholesterol; therapeutic target for stroke prevention	[133]
*IL6-R*	Interleukin-6 receptor	Reduced function linked to lower stroke risk	Anti-inflammatory pathway modulated via Asp358Ala variant	[146]
*F11*	Encodes coagulation factor XI	Increased levels linked to higher cardioembolic stroke risk	Enhances thrombin generation; potential antithrombotic drug target	[147,148]

## Data Availability

No new data was created or analyzed in this study. Data sharing is not applicable to this article.
